# Micro Water Flow Measurement Using a Temperature-Compensated MEMS Piezoresistive Cantilever

**DOI:** 10.3390/mi11070647

**Published:** 2020-06-30

**Authors:** Romain Pommois, Gaku Furusawa, Takuya Kosuge, Shun Yasunaga, Haruki Hanawa, Hidetoshi Takahashi, Tetsuo Kan, Hisayuki Aoyama

**Affiliations:** 1 École Nationale Supérieure de Mécanique et des Microtechniques, 26 Rue de l’Épitaphe, 25000 Besançon, France; romain.pommois@gmail.com; 2 Department of Mechanical and Intelligent Systems Engineering, Graduate School of Informatics and Engineering, The University of Electro-Communications, 1-5-1 Chofugaoka, Chofu-city, Tokyo 182-8585, Japan; gaku@ms.mi.uec.ac.jp (G.F.); kosuge@ms.mi.uec.ac.jp (T.K.); hanawa@sys.aolab.mce.uec.ac.jp (H.H.); aoyama@mce.uec.ac.jp (H.A.); 3 Department of Mechano-Informatics, Graduate School of Information Science and Technology, The University of Tokyo, 7-3-1 Hongo, Bunkyo-ku, Tokyo 113-8656, Japan; yasunaga@hybrid.t.u-tokyo.ac.jp; 4 Department of Mechanical Engineering, Faculty of Science and Technology, Keio University, 3-14-1 Hiyoshi, Kouhoku-ku, Yokohama, Kanagawa 223-8522, Japan; htakahashi@mech.keio.ac.jp

**Keywords:** microelectromechanical system (MEMS) cantilever-type force sensor, temperature compensation, microflow measurement

## Abstract

In this study, we propose a microelectromechanical system (MEMS) force sensor for microflow measurements. The sensor is equipped with a flow sensing piezoresistive cantilever and a dummy piezoresistive cantilever, which acts as a temperature reference. Since the dummy cantilever is also in the form of a thin cantilever, the temperature environment of the dummy sensor is almost identical to that of the sensing cantilever. The temperature compensation effect was measured, and the piezoresistive cantilever was combined with a gasket jig to enable the direct implementation of the piezoresistive cantilever in a flow tube. The sensor device stably measured flow rates from 20 μL/s to 400 μL/s in a silicon tube with a 2-mm inner diameter without being disturbed by temperature fluctuations.

## 1. Introduction

With the advancement of microfluidics, technology to continuously measure microfluidic flow is significantly important in various fields [1,2,3,4,5]. Among these technologies, microelectromechanical system (MEMS) flow sensors are suitable for microfluidics because they can measure minute flows, and their small sensor size permits a direct integration into small fluidic channels [6]. Thermal-type sensors are the mainstream of small MEMS flow sensors, which involve placing a hot wire in a flow and measuring the flow rate based on the amount of heat carried by the flow [7,8,9,10,11]. A thermal MEMS sensor is highly sensitive and can be miniaturized. However, the heat generated on the wire could damage solutions containing biological samples. In contrast, measuring the flow rate based on the drag force of the fluid using a MEMS force sensor is a promising technique because in principle the sensor does not interact with the biological sample [12,13,14,15,16,17,18,19,20]. For the sensitive flow rate measurement, a MEMS force sensor-type flowmeter involves placing a cantilever in the flow path and detecting its strain caused by the drag force. Some MEMS force sensors detect the strain in an optical way; a shift of the reflected laser spot position or deflected cantilever’s optical image itself were used to detect the flow [13,14]. The other sensors mainly detect the strain as a resistance change electrically by means of a piezoresistor formed on the cantilever, this approach having the advantage of facilitating the miniaturization of the sensor configuration. For example, paddle-like shaped cantilevers equipped with piezoresistors on the cantilever roots were employed to detect the force by the flow [12,18]. Self-bended cantilevers were also employed to effectively detect the flow force [15,18]. In addition to the simple flow rate measurement, piezoresistive cantilever typed flow sensors were applied for viscosity sensing [19] or even a bio-logging application of seabirds [16]. The addition of some structures, such as bristles [17] and pillars [20], was performed to improve the sensor performance. Among them, since the piezoresistance also changes according to the temperature conditions around the sensor, temperature compensation is required to precisely measure the flow rate. Conventionally, a dummy piezoresistor on a thick sensor board has often been employed [12,15,20,21,22,23]. However, the temperature environment would be different between the cantilever protruding into the space and the piezoresistor on the substrate; thus, constructing a dummy piezoresistor in the form of a cantilever for accurate flow sensing is desirable.

In this study, we propose a MEMS force sensor for microflow measurement, which is equipped with a flow sensing piezoresistive cantilever and a dummy piezoresistive cantilever, acting as a temperature reference. In the field of molecular sensing, dummy resistors are constructed in the form of cantilevers to remove noises or offsets other than measurement signals [24,25,26,27]. In particular, we have observed that offset on the output signal can significantly be reduced using a dummy cantilever for compensation when a force sensing MEMS cantilever was used under microscopy; it largely improved force sensing preciseness [27]. Expanding this idea, the dummy cantilever was also fabricated in the form of a thin cantilever so that the temperature environment of the dummy cantilever was almost identical to that of the sensing cantilever; the difference between the cantilevers was only the length of the area where the drag force was applied. To verify the practicality of the proposed sensor, the temperature compensation effect was measured, and the piezoresistive cantilever was combined with a gasket jig to enable the direct implementation of the piezoresistive cantilever in a flow tube. With this configuration, a sensor device can stably compensate for the temperature effect on the resistance change; a reference cantilever that did not have measure flow rates from 20 μL/s to 400 μL/s was realized. This paper also describes in detail the strain-resistance change characteristics, the temperature compensation performance of a prototype piezoresistive cantilever-type sensor, the fabrication of the sensor and the gasket jig design to support the sensor. Finally, the validity of the sensor for the flow measurement is described.

## 2. Measurement Principle

**Figure 1:**
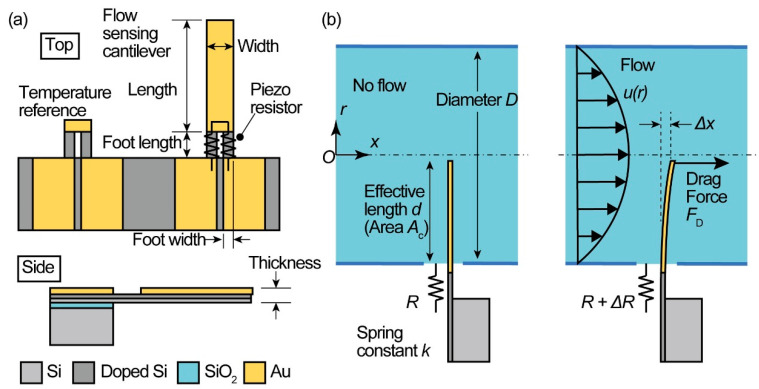
Proposed sensor configuration. (**a**) Design of the piezoresistive cantilever, and (**b**) water flow rate measurement.

In this research, a MEMS piezoresistive cantilever was employed to measure the microflow rate. The MEMS piezoresistive cantilever sensor used in this research is shown in Figure 1a. The piezoresistive cantilever-type sensor is composed of two parts: a flow sensing cantilever and a dummy temperature reference cantilever. These two cantilevers are formed on the same substrate.

Piezoresistors were formed at the foot of the cantilevers with a resistance *R*, and their resistance changed by Δ*R* when strain was applied to them. The cantilever area, except for the foot, was covered with the Au conducting film so that only the strain on the foot could be measured. Since the baseline resistance of the flow sensing cantilever changes depending on the temperature of the sensor, the dummy temperature reference cantilever was formed beside the sensing cantilever to compensate for the temperature effect on the resistance change; the reference cantilever did not have a cantilever body and only piezoresistors at its root. The reference cantilever also protruded from the substrate and was immersed in the flow channel so that it was in an identical temperature environment as the flow sensing cantilever.

A water flow measurement was performed by placing the flow sensing cantilever in a water tube, as shown in Figure 1b. Cylindrical coordinates (*r*, *x*) were used to define the model. Since we regard a cross section of the water flow tube to be circular with an inner diameter of *D*, the flow velocity profile *u*(*r*) is a function of the position *r* from the center of the tube and becomes:

(1) $$u\left(r\right)=2\left(1-\frac{r^{2}}{{\left(\frac{D}{2}\right)}^{2}}\right)u_{\mathrm{ave}},$$

where *u*_ave_ is the average flow velocity. *u*_ave_ can also be expressed as *u*_ave_ = *Q*/*A*, where *Q* is the flow rate, i.e., the amount of volume that flows per unit time, and *A* is the cross-sectional area of the tube, which is equal to π(*D*/2)^2^.

When no flow occurs, the resistance of the cantilever is maintained at the initial resistance *R*. When water flow occurs, the cantilever is subject to the drag force *F*_D_. If we take *u*_ave_ as a representative flow velocity and regard the drag force *F*_D_ as a concentrated force at the tip of the cantilever, as shown in Figure 1b, then the drag force is expressed as:

(2) $$F_{D}=\frac{1}{2}\rho C_{D}A_{c}u_{\mathrm{ave}}^{2},$$

where *ρ* is the density of the fluid (kg/m^3^), *C*_D_ is the cantilever drag coefficient (dimensionless), and *A*_c_ is the concerned cantilever area (m^2^). The drag force bends the cantilever in the *x*-direction by a deflection of Δ*x*, which gives rise to strain at the root of the cantilever, and the resistance of the cantilever also changes to *R* + Δ*R*. Here, we simplified the drag force which is a distributed load as a concentrated force at the tip of the cantilever. As shown in Figure 1b, the cantilever is subjected to a parabolic distribution of the distributed load. This acts as a bending moment on the root of the cantilever. If we calculate the contribution of the distributed load on the bending moment at the root of the cantilever by integrating it, the total bending moment at the root can be regarded as being produced by a point concentrated force at the cantilever tip. In this case, the relationship between the flow velocity *u*_ave_ and the produced moment at the root, which is equivalent to the fractional resistance change Δ*R*/*R* of the piezoresistor, becomes a one-to-one correspondence. The velocity of the flow *u*_ave_ can thus be determined with a model of a single point load. In what follows, the drag force is regarded as a point load, and the one-to-one correspondence coefficients will be obtained experimentally. Here, we denote the spring constant of the cantilever by *k*, which is defined as the ratio between the force *F* acting on the cantilever tip and the *x*-directional deflection Δ*x*: *F* = *k*Δ*x*. The fractional resistance change Δ*R*/*R* per unit *x*-directional deflection Δ*x* is denoted as *S*_d_: Δ*R*/*R* = −*S*_d_Δ*x*. The relationship between the fractional resistance change and the average flow velocity can thus be expressed as:

(3) $$\frac{\Delta R}{R}=-\frac{S_{d}}{k}\frac{1}{2}\rho C_{D}A_{c}u_{\mathrm{ave}}^{2}.$$

Since the parameters, except for Δ*R*/*R* and *u*_ave_, are constant in an actual measurement environment, the flow velocity can be measured by experimentally determining these constant parameters.

## 3. Fabrication and Assembly

The design parameters of the piezoresistive cantilever are as follows. We designed the sensor so that it can be embedded in a water tube with a 2-mm inner diameter, which was typically used in our microfluidic measurements. To verify the performance of the sensor, the target water flow rate was chosen to be ~100 μL/s, which is usually employed for cellular manipulation in our group [28]. Moreover, the cantilever body was required to not inhibit the water flow. The flow sensing cantilever dimensions were thus chosen to be 1074 μm in length, 256 μm in width, 250 μm in foot length, and 96 μm in foot width, so that the cantilever had an appropriate stiffness to measure the target flow rate and was adequately smaller than the tube cross section. For the temperature reference cantilever, the foot was the same as that for the flow sensing cantilever, but it did not have a cantilever body part attached. The thicknesses of these cantilever-type sensors were uniformly 5 µm. The main structure of the cantilever was made of crystalline N-type silicon (Si). Assuming that the Young’s modulus of Si is 169 GPa [29], the cantilever spring constant *k* for a single point load at the tip was calculated to be 0.56 N/m by a finite element method (FEM) simulation, which was used in the calculation of Equation (3) below.

**Figure 2:**
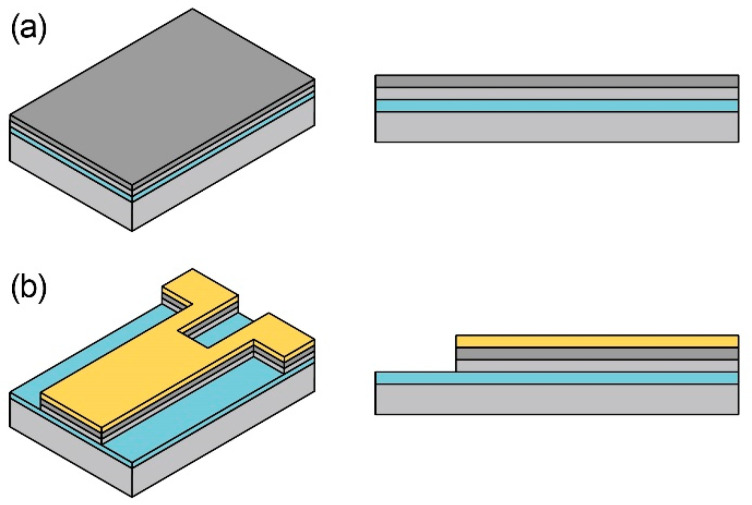

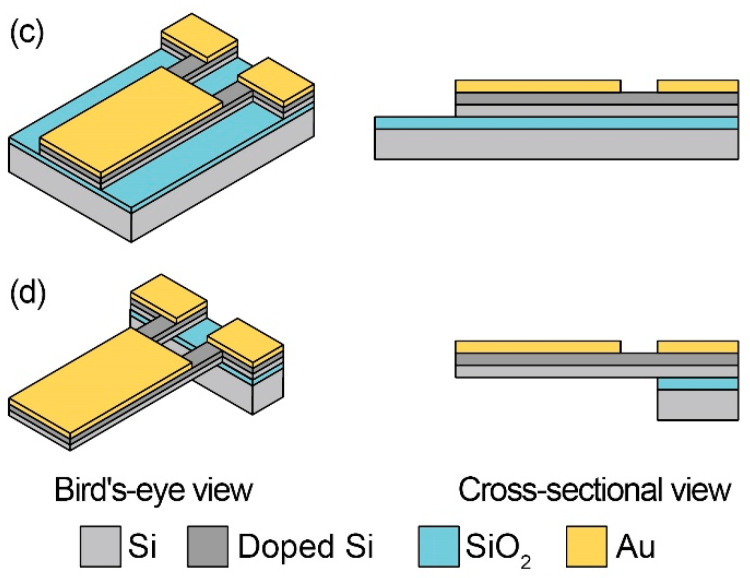

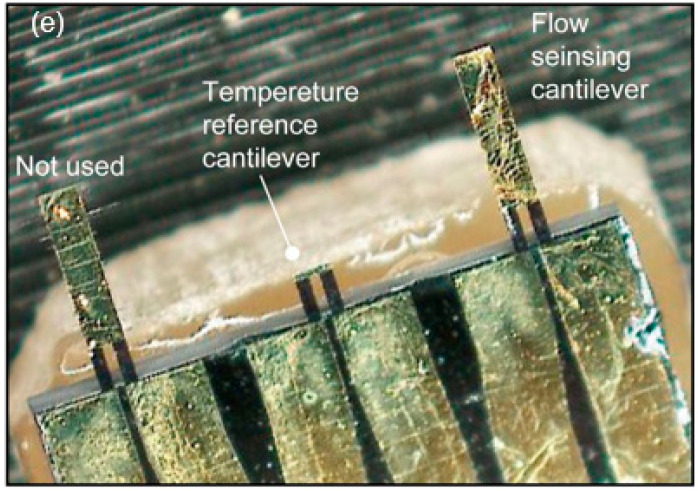
Microelectromechanical system (MEMS) fabrication process and photograph of the fabricated cantilever-type sensor. (**a**) To form the N-type piezoresistor on a 5/2/300 μm silicon-on-insulator (SOI) wafer, (**b**) to deposit an Au layer on the device Si layer and etch the Au/ device Si layers, (**c**) to pattern the Au layer, and (**d**) etching the handle Si/SiO2 layers; (**e**) a photograph of the fabricated cantilever.

The fabrication process of the piezoresistive cantilever is shown in Figure 2a–d. The cantilever device was fabricated by a standard bulk micromachining process. In the process, a p-type silicon-on-insulator (SOI) wafer (5 μm device Si layer, 2 μm SiO_2_ layer, and 300 μm handle Si layer) was used as the starting material. The fabrication process is described in previous studies in detail [17]. First, an n-type piezoresistor was formed on the surface of the device Si layer by rapid thermal diffusion (Figure 2a). The doping concentration was approximately 10^20^ cm^−3^. Second, an Au layer was deposited on the doped Si surface and patterned to form electrodes (Figure 2b). Au was also deposited on the cantilever itself so that only the cantilever root with two legs worked as a piezoresistor. Then, the device Si layer was also etched with inductively coupled plasma reactive ion etching (ICP-RIE). The Au layer was etched again (Figure 2c). The handle Si layer was etched from the backside with ICP-RIE, and the SiO_2_ layer was etched with HF vapor (Figure 2d). In this way, the free-standing MEMS piezoresistive cantilevers were fabricated. The resistance of both the fabricated flow sensing and temperature reference cantilevers was approximately 1 kΩ. The fabricated cantilevers bonded on a printed circuit board are shown in Figure 2e. The doping impurity concentration using the same thermal diffusion process has been reported elsewhere to be around 10^20^ cm^−3^ [30].

**Figure 3:**
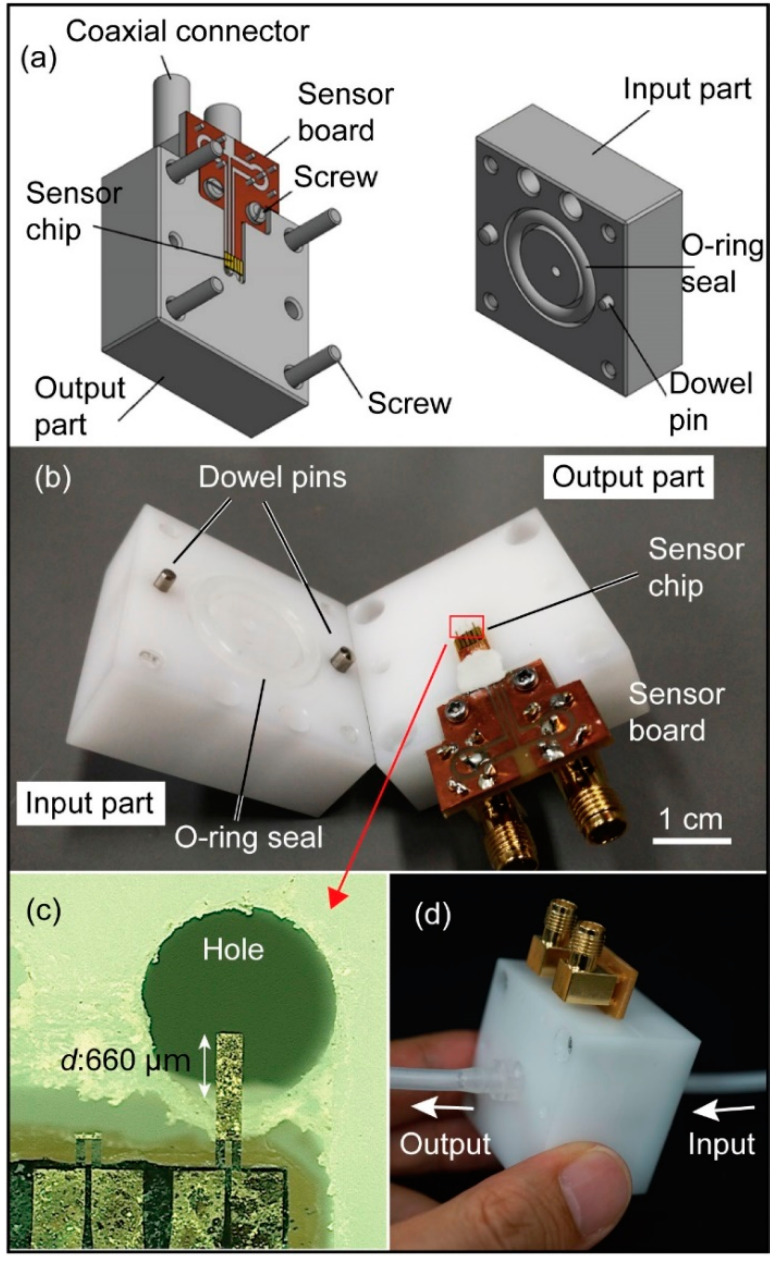
A cantilever of the MEMS force sensor-type flowmeter integrated with the gasket jig. (**a**) 3D design of the sensor assembly, (**b**) mechanical jig parts composed of input and output white POM parts and an attached sensor board for sensor assembly, (**c**) magnified image of the cantilever tip placed over a water flow hole of the output mechanical part, and (**d**) assembled sensor component connected to tubes.

The gasket jig was designed and fabricated so that the cantilever was embedded in the tube flow. The gasket jig was composed of two main parts: an input part where water flows from a tube to the device and an output part where the water flows from the device to a tube. Figure 3a shows a three-dimensional CAD schematic of the gasket jig. These two parts were made of thermoplastic polyoxymethylene (POM) because it offers many favorable physical properties for flow measurement purposes: high stiffness, dimensional stability, nonconductivity, and ethanol resistance. Holes for the water flow were formed at the middle of each mechanical part. The hole on the output part is located under the sensor hip in Figure 3a. Tubes were directly connected to the parts via luer fittings. In between the two parts there was the sensor board (Figure 3a,b), composed of the sensor chip and its printed circuit board support. The sensor chip was attached to the board support with epoxy glue. The sensor board was then screwed to the output part. The sensor chip was electrically connected to the printed circuit board by wire bonding. The electrical signals were transmitted to the measurement bridge circuit via SMA coaxial cables. The flow sensing cantilever was aligned on the output part so that it protruded into the hole by approximately 660 μm, corresponding to the effective length *d* in Figure 1b, as shown in Figure 3c. To achieve water-tightness, an O-ring seal was placed around the hole on the input part, as shown in Figure 3a,b). Note that the dummy temperature reference cantilever was also placed inside the O-ring and immersed in water. Clay paste was placed around the neck of the sensor board to prevent water leakage around the sensor board, corresponding to the white area between the sensor chip and the sensor board in Figure 3b. Moreover, to ensure that the water flow holes were aligned straight, dowel pins were used to set the two mechanical parts in position while assembling them tightly together with screws, as shown in Figure 3b. The assembled sensor configuration is shown in Figure 3d, and in this way, the proposed flow sensor was inserted into the water flow tubing system.

## 4. Cantilever Response Measurement

**Figure 4:**
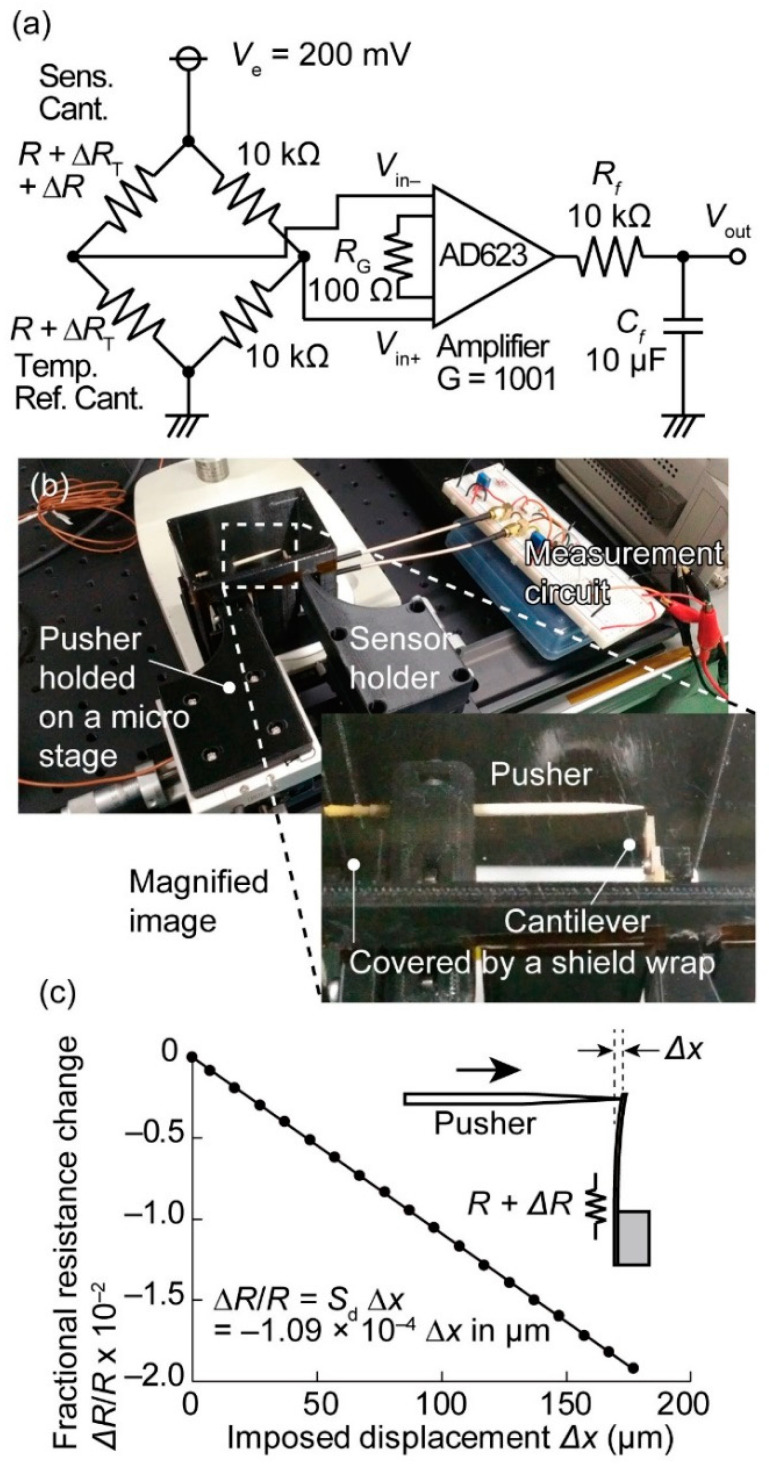
Measurement of the electrical characteristics of the MEMS cantilever-type force sensor. (**a**) Electrical circuit diagram for the strain measurement, (**b**) an experimental setup for pushing the cantilever, and (**c**) the displacement and measured fractional resistance change.

The resistance change of the piezoresistive cantilever was measured using a Wheatstone bridge circuit, as shown in Figure 4a. A fractional resistance change induced by a temperature change was denoted as Δ*R*_T_. In the fabricated piezoresistor, the amount of temperature dependence is reported to be 2448 ppm/°C [30]. The flow sensing piezoresistive cantilever was placed at the left upper part of the bridge. To eliminate the temperature variation effect on the water flow sensing, a temperature compensation sensor was placed at the left lower part of the bridge. If the resistance at the left lower part does not have temperature responsibility, unlike the reference piezoresistive cantilever, then the output becomes:

(4) $$\Delta V=V_{\mathrm{in}+}-V_{\mathrm{in}-}\sim V_{e}·\frac{\Delta R+\Delta R_{T}}{4R},$$

which is significantly susceptible to temperature variations. If the temperature reference piezoresistive cantilever is used, then the bridge output becomes:

(5) $$\Delta V\sim V_{e}·\frac{\Delta R}{4R}{,}_{\;}$$

where the temperature effect on the output signal can mostly be eliminated. The bridge output was amplified 1.00 × 10^3^ times by an instrumentation amplifier (Analog Devices, AD 623, Norwood, MA, USA). In addition, since 50 Hz power supply noise was found in the measured signal, an RC lowpass filter was inserted between the amplifier and the output port so that only the slowly changing signal was measured.

Furthermore, the fractional resistance change Δ*R*/*R* per unit *x*-directional deflection Δ*x* of the cantilever *S*_d_ was measured. The deflection was applied by pushing the tip of the flow sensing cantilever in the *x*-direction using a pointed probe. The deflection sensing was performed under a microscope. The detailed pushing environment is shown in a magnified image of Figure 4b. Although it is difficult to see the cantilever, the pusher pointed the tip of the cantilever. To eliminate fluctuations from the air, the cantilever and the point probe were enclosed by a shielding box, and the top side of the box was covered with a plastic wrap. The x-directional displacement of the point probe was supplied by a manual micrometer, and the contact between the cantilever and the probe was observed by a microscope. Δ*R*/*R* was plotted with respect to the x-directional deflection Δ*x* in Figure 4c. The cantilever deflection Δ*x* and Δ*R*/*R* presented a clearly linear relationship; the linear relationship was not distorted, even for a deflection as large as 180 μm. *S*_d_ was calculated to be 1.1 × 10^2^ m^−1^ from the slope of the relationship, which is coherent with a previous report on the same device configuration [17]. The linearity was newly confirmed to be maintained for larger deflections; in the previous report, the linearity was examined only for deflections as large as 5 μm. Since the spring constant of the cantilever was 0.56 N/m, the maximum deflection in this measurement corresponded to an approximately 100 μN force input.

## 5. Temperature Compensation Effect

**Figure 5:**
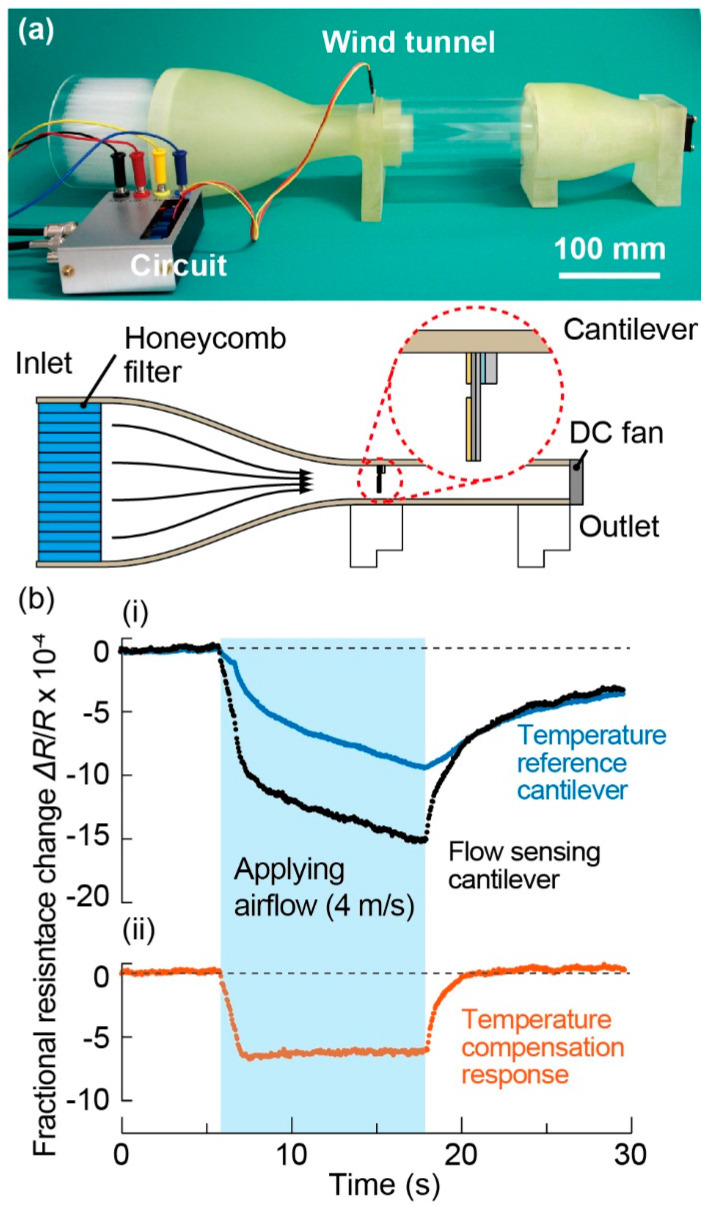
Temperature compensation effect. (**a**) A photograph of a wind tunnel used to verify the temperature compensation effect. (**b-i**) Fractional resistance changes of the flow sensing cantilever and temperature reference cantilever when applying an airflow velocity of 4 m/s in an airflow tunnel. (**b-ii**) Temperature compensation response calculated using both responses.

The temperature compensation effect was investigated by measuring the amplifier output with and without the reference cantilever. In this experiment, the responses of the flow sensing cantilever and the temperature reference cantilever were separately measured using two different bridge circuits based on the one-gauge method, so that each response was separately measured. *V*_e_ = 1 V was applied to the bridge circuit. For the one-gauge method, a static resistance was placed at the left lower part of the bridge (Figure 4a). In the experiment, we applied airflow instead of water flow to the cantilevers using an airflow tunnel at room temperature [16]. Figure 5a shows a photograph of the airflow tunnel, most of whose body was constructed using a 3D printer. The detailed explanation is given in a reference [16]. When an airflow velocity of 4 m/s was applied, both outputs of the cantilevers abruptly changed, as shown in Figure 5b-i. Even when the airflow velocity became stable, the outputs still gradually decreased, which can be attributed to decreases in the environmental temperature and the cantilever temperature. After stopping the airflow, both outputs gradually returned to the initial values. The temperature compensation response was calculated using both outputs, as shown in Figure 5b-ii. The calculated response corresponded to the response obtained via the two-gauge method using both cantilevers. The obtained results indicated that the temperature change due to flow may cause a significant signal noise, and that the temperature change effect can largely be eliminated by using the proposed temperature compensation sensor configuration. It is noted that the reference cantilever also receives and presents a response induced by the airflow force in addition to the temperature response. The calculation based on the procedure in [31] revealed that the amplitude of response by the airflow force measured with the reference cantilever is only 2% of that of the sensing cantilever. We therefore considered that, in this experiment, the force response of the reference cantilever was negligible and the effectiveness of the temperature compensation was valid. It should also be noted that, in this case, the temperature compensation effect was investigated using the wind tunnel and a laminar air flow. In an actual water flow environment, thermal phenomena such as convection heat transfer may occur and disturb the temperature environment of both sensing and reference cantilevers. For practical purposes, the uniformity between these two cantilevers should be investigated in situ in the future.

## 6. Water Flow Measurement

**Figure 6:**
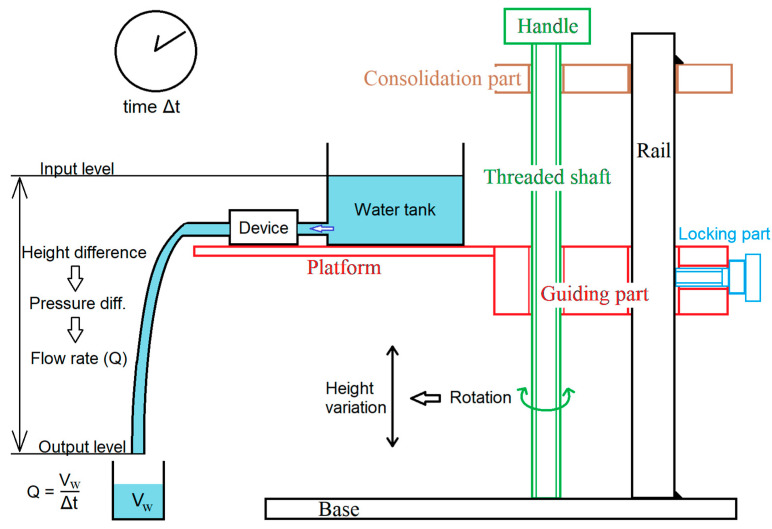
Height adjustment setup to change the flow rate.

Microflow rate detection experiments were performed. As a driving force of the microflow, gravity was adopted. The pressure resulting from the height difference between the wafer surface of a tank and a tube exit provided a stable water flow (Figure 6). To keep the water surface height constant and remove fluctuations in the flow rate, a large water tank was used. The height difference could be changed by rotating the threaded shaft. It translated a guiding part and a platform, on which the water tank located. The locking part fixed the height of the guiding part. In the experiment, water flow disturbance due to the insertion of the sensor was not observed. The flow rate *Q* was calculated by dividing the quantity of water that flowed from the water tube outlet by the flow time, and the average flow velocity *u*_ave_ was calculated by *Q*/*π*(*D*/2)^2^, where *D* was 2 mm. The flow rate was altered by changing the height of the water tank. The assembled sensor was inserted in the water tube, and the amplified output signal was measured by an oscilloscope.

**Figure 7:**
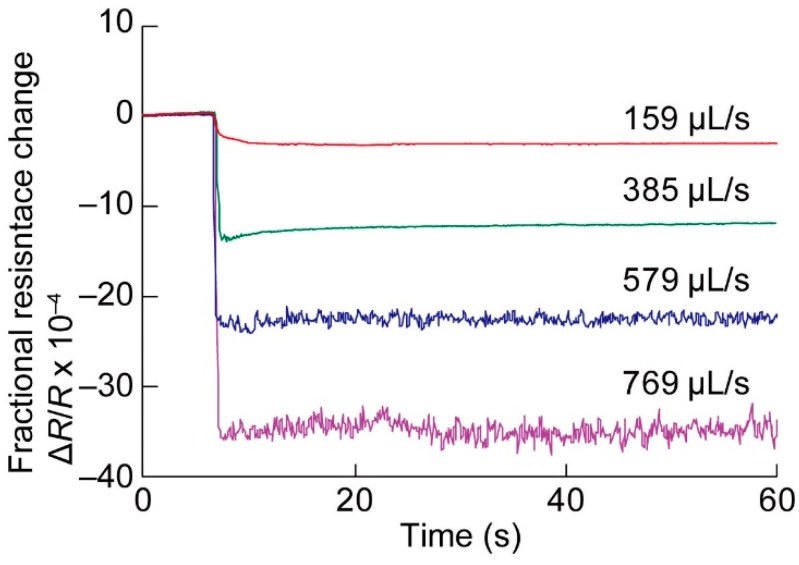
Water flow rate measurement. Time course data of the amplifier output voltage for different water flow rates.

**Figure 8:**
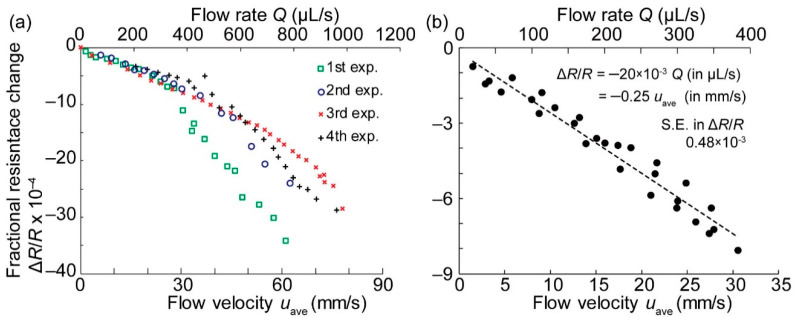
Water flow rate measurement. (**a**) Fractional resistance change with the flow rate/velocity, and (**b**) magnified relationship between the flow rate/velocity and the fractional resistance change at low flow rates.

The data obtained for four different flow rates were calculated in the form of Δ*R*/*R*, as shown in Figure 7. The voltage *V*_e_ applied on the bridge circuit was kept as small as 200 mV to avoid electrolysis and the destruction of the cantilever. With the aid of thermal compensation, the output waveform was uniform without the effect of temperature fluctuations. The output voltage tended to increase as the flow rate *Q* increased. At lower flow rates such as 159 and 385 μL/s, the resistance changes were almost stably constant. On the contrary, at the higher flow rates, 579 and 769 μL/s, noise on the resistance change appeared, which can be attributed to the vortex around the cantilever or the flutter of the cantilever. The time course data output was averaged, and the fractional resistance changes Δ*R*/*R* were plotted with respect to both the flow rate *Q* and the average flow velocity *u*_ave_ in Figure 8a. The results of four experiments are shown. Since the piezoresistive cantilever was formed on an N-type Si substrate, the resistance decreased when the cantilever was subjected to tensile stress. Thus, the fact that Δ*R*/*R* monotonically decreased with an increasing flow rate was a reasonable result. In the lower flow rate range, Δ*R*/*R* was almost linear with *Q* and *u*_ave_. In the higher flow rate range, particularly over 400 μL/s, the relationship between *Q* and Δ*R*/*R* deviated from the linear relationship. These behaviors can be attributed to the fact that Δ*R*/*R* is a quadratic function of *u*_ave,_ as expressed in Equation (3). At a flow rate lower than 400 μL/s, the data presented repeatability. At flow rates higher than 400 μL/s, the fluctuations between the four experiments were large. The increase in fluctuations can be attributed to the disturbance of flow around the cantilever at the higher flow rates, which can be partly seen in the raw data in Figure 8a, where the amplitude of noise in the graph apparently increased for flow rates over 579 μL/s. In addition, the fourth experiment presented a large deviation from the other three experiments. This can be attributed to the experimental setup limitation. In this setup, we employed a silicone tube easily bended due to the inertial force exerted by a fast flow, which would alter flow resistance during the experiment. An improvement of repeatability can be realized by using metal rigid tubes in the setup.

We therefore obtained data focusing on the flow range below *Q* < 400 μL/s, as shown in Figure 8b. When the flow rate was low, Δ*R*/*R* was approximately linear with the flow rate *Q.* However, in Equation (3), Δ*R*/*R* should have been a quadratic function with respect to the flow velocity *u*_ave_. This linear behavior can be interpreted as follows. At a low Reynolds number, as in this low flow velocity case, the drag coefficient *C*_D_ is inversely proportional to the flow velocity *u*_ave_ [32]. Therefore, in this low flow velocity range, Δ*R*/*R* in Equation (3) becomes linear with the flow velocity, instead of quadratic. These repeatable results indicated that the proposed sensor could be used for measuring low flow velocities. However, around a zero flow rate, the sensor precision and artifacts may limit the measurable flow rate. We therefore evaluated the lower limit of the measurable flow rate to be 20 μL/s, which corresponded to the minimum measured flow rate in the experiment in Figure 8b. This flow rate can be converted to the flow velocity, and the flow rates from 20 μL/s to 400 μL/s correspond approximately to the flow velocities *u*_ave_ from 1.5 to 30 mm/s. The fitted line in Figure 8b was expressed as Δ*R*/*R* = −20 × 10^−3^ *Q* (in μL/s unit) or Δ*R*/*R* = −0.25 *u*_ave_ (in mm/s unit). The standard error against the fitted line with respect to Δ*R*/*R* was 0.48 × 10^-3^. This flow velocity range can be measured with the proposed device by directly connecting it to a flow tube. Moreover, the drag coefficient *C*_D_ of the cantilever for *u*_ave_ = 20 mm/s was calculated to be ~76 from Equation (2). The Reynolds number at this flow velocity was approximately 5, taking the cantilever width as the representative length and *u*_ave_ as the representative velocity so that the water flow was viscous. According to the literature, the drag coefficient of a square plate around this Reynolds number becomes ~10 [32] so that the calculated *C*_D_ is several times larger than that in the literature. The flow velocity of the small tube was measured with a cantilever with almost the same scale as the tube. Since the cantilever might interrupt the water flow, the cantilever received a larger inertial force than the measurement conducted in a free space, making the drag coefficient larger. Although the obtained experimental results provide a larger drag coefficient for the cantilever, the difference is of the same order, so that it can be concluded that a low flow rate can be measured with the proposed device. The size reduction of the cantilever will provide a better flow sensing device, which is attainable with MEMS technology. In addition, the cantilever in this study is fabricated using a 5-μm-thick Si membrane, and further thinning of the cantilever is easily attainable, for example to a several hundreds of nm-thick cantilever [30,33,34,35,36,37,38,39]. Since the force sensitivity of the cantilever type force sensor will improve with the cube of the thickness, a further reduction of the measurable flow rate will be possible. The piezoresistive cantilever type force sensor will provide a simple and accurate way of sensing the flow rate in microfluidics.

## 7. Conclusions

In this study, we proposed a MEMS force sensor for a microflow measurement. The MEMS force sensor was equipped with a flow sensing piezoresistive cantilever and a dummy piezoresistive cantilever, which acted as a temperature reference. The dummy cantilever was designed to be a protruding thin cantilever from the substrate so that the temperature environment of the dummy sensor became identical to that of the sensing cantilever. The temperature compensation effect was evaluated, and the temperature effect was almost eliminated based on the data obtained in a wind tunnel. The piezoresistive cantilever was then combined with a gasket jig to enable the direct incorporation of the piezoresistive cantilever into a flow tube with an inner diameter of 2 mm. The sensor device was able to stably measure flow rates from 20 μL/s to 400 μL/s without a temperature fluctuation effect. Since the drag coefficient *C*_D_ calculated from the measured data was almost consistent with a previous study, the validity of the measured flow rate was confirmed. Compared with previous piezoresistive type flow sensors [12,15,18], the proposed sensor is equipped with a cantilever-typed thermal compensation element, which will be more effective in eliminating the thermal offset on the sensor output. The proposed sensor can be fabricated with an inert material that does not interact with biological samples so that it does not damage biological fluid. Moreover, since the proposed sensor can be inserted in tandem into a fluid tube, these features make it suitable for microfluidic systems.
